# Evaluation of the rabbit liver by direct portography and contrast-enhanced computed tomography: anatomical variations of the portal system and hepatic volume quantification

**DOI:** 10.1186/s41747-017-0011-8

**Published:** 2017-06-29

**Authors:** María Páramo, Paula García-Barquin, Eva Santa María, José Miguel Madrid, Meylin Caballeros, Alberto Benito, Bruno Sangro, Mercedes Iñarrairaegui, José Ignacio Bilbao

**Affiliations:** 1 Clínica Universidad de Navarra, Avenida Pio XII, 36, 31008 Pamplona, Navarra Spain; 2 Program of hepatology, CIMA, CIBEREHD, Clínica Universidad de Navarra, Avenida Pio XII, 36, 31008 Pamplona, Navarra Spain; 3 IdisNA, Clínica Universidad de Navarra, Avenida Pio XII, 36, 31008 Pamplona, Navarra Spain; 4 CIBEREHD, IdisNA, Liver Unit. Clínica Universidad de Navarra, Avenida Pio XII, 36, 31008 Pamplona, Navarra Spain

**Keywords:** New Zealand white rabbits, Portal system, Anatomy, Portography, Contrast-enhanced CT

## Abstract

**Background:**

The study was aimed at: (1) describing the incidence of anatomic variations of the portal system in the rabbit using direct portography; and (2) estimating the liver volume and caudate lobe volume by using contrast-enhanced computed tomography (CECT) in the same animal model.

**Methods:**

Forty-six New Zealand white rabbits were included. All of them underwent direct portography and unenhanced CECT. Conventional liver rabbit portal system anatomy (type 1) consisted of the bifurcation of the main portal vein (MPV) into the right portal vein (RPV) and left portal vein (LPV), which subsequently divided into medial left portal vein and lateral left portal vein. Trifurcation of the LPV was considered type 2. The LPV that divides into four smaller branches was classified as type 3. Other configurations of the portal system, including particular cases of MPV branching, were grouped as type 4. Liver lobes were manually segmented.

**Results:**

The incidence of each type of portal system anatomy was: type 1, 67.4%; type 2, 15.2%; type 3, 13.0%); and type 4, 4.3%. The mean volume of the caudate lobe was 19.1 ml ± 5.7 ml and of the cranial lobes it was 66.7 ml ± 13.7 ml, and the total liver volume was 85.7 ml ± 16.7 ml.

**Conclusions:**

In New Zealand white rabbits, type 1 is the prevalent type of portal system, liver volume is about 86 ml, and the caudate and cranial lobes are separated. This information could be important when planning experimental rabbit liver procedures.

## Keypoints

- Direct portography provides information about rabbit liver portal anatomy and its variations
- The caudate and cranial lobes are separated, allowing both areas to be studied independently
- Manual delineation of the liver lobes by contrast-enhanced computed tomography provides volumetric information

## Background

Rabbits are often used as models in research, because these animals have several morphological similarities in the hepatic vascular system to humans [1]. Moreover, the rabbit VX2 tumour model is commonly adopted in experimental oncology because it allows the preclinical evaluation of several embolic agents and endovascular devices [2]. Any experimental development on surgical or interventional procedure performed on the liver of a rabbit requires in-depth knowledge of the anatomy and of the anatomical variations of this animal model.

**Fig. 1:**
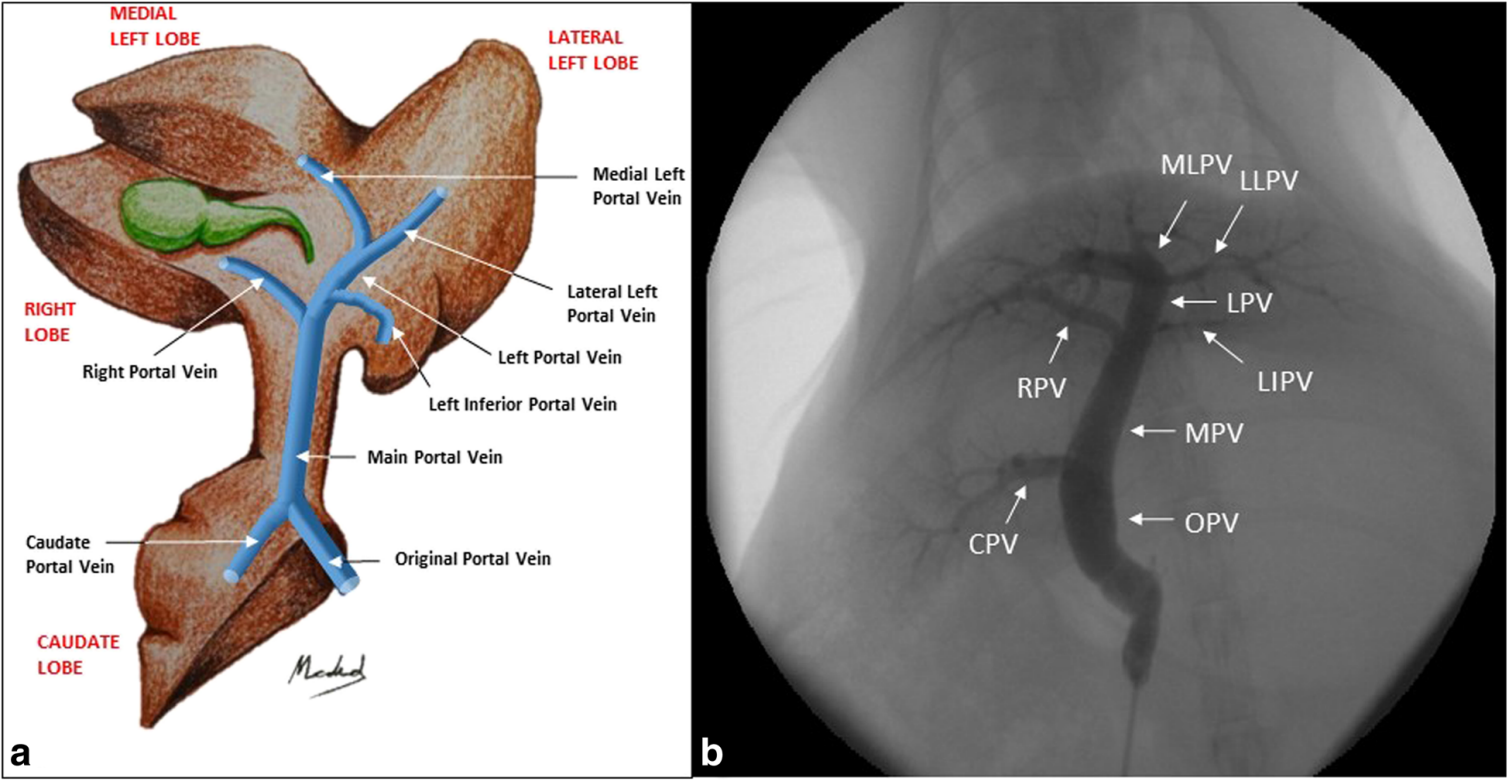
**a** Anatomy of rabbit liver. **b** Direct portography of a conventional rabbit portal system anatomy (anatomic variation type 1). The original portal vein (*OPV*) divides into the main portal vein (*MPV*) and caudate portal vein (*CPV*). The MPV then bifurcates into the right portal vein (*RPV*) and left portal vein (*LPV*). The left inferior portal vein (*LIPV*) originates from the LPV. Then the LPV bifurcates into segmental branches, the medial left portal vein (*MLPV*) and the lateral left portal vein (*LLPV*)

The liver anatomy of the rabbit has some relevant differences compared to a human liver. Rabbit liver is subdivided into four main lobes [3]. These are the caudate lobe and three cranial lobes, comprising the right, medial left and lateral left lobes, each of them supplied by branches of the portal venous system (Fig. 1a). An important characteristic of the rabbit liver is that the caudate and the cranial lobes are separated, thus allowing both areas to be studied independently. Focusing on the portal venous system, the most common anatomical pattern, called “conventional”, is the presence of an original portal vein (OPV), which is formed by the confluence of the mesenteric and the gastrosplenic veins [4]. The OPV is subdivided into the caudate portal vein (CPV) and the main portal vein (MPV) (Fig. 1a, b). The CPV is located to the right side of the OPV and the MPV bifurcates into the right portal vein (RPV) and left portal vein (LPV), which further divides into the medial left portal vein (MLPV) and lateral left portal vein (LLPV). The left inferior portal vein (LIPV) is defined as an accessory branch originating from the LPV, the MPV or both [4].

There have only been a few studies published detailing the radiological anatomy of the rabbit portal system, and these included a limited number of animals [3, 4]. Larger series are needed for further information about the standard rabbit liver anatomy and its variants as a suitable basis for experimental studies.

This study was conducted to determine the most common (conventional) portal system anatomy and its variations using direct portography and to quantify the liver volume using contrast-enhanced computed tomography (CECT) on a large series of rabbits.

## Methods

### Animals

All applicable institutional and/or national guidelines for the care and use of animals were followed. Forty-six New Zealand white female rabbits weighing around 3 kg (range 2.68 − 3.55 kg) were included. This is an investigation in the context of an already established experimental study planned for other purposes. All rabbits first underwent basal CECT followed by direct portography.

### CECT acquisition protocol

All examinations were performed with a 64-MDCT scanner (Somatom Sensation, Siemens Medical Systems, Erlangen, Germany). Examination parameters were 64 × 0.6 mm collimation, 1.4 mm/s table feed, 2 mm section thickness, 1.5 mm reconstruction interval, 80 kV, 65 mA and 0.5 s rotation time. Rabbits were placed in the supine position and were sedated by intramuscular injection of 100 mg/ml ketamine (Imalgene®, Merial) and 1 mg/ml of medetomidine (Domtor®, Esteve veterinaria). After unenhanced scan acquisition, a contrast-enhanced scan was performed 15 s (arterial phase), 30 s (portal phase), 45 s (venous phase), and 60 s (late venous phase) after intravenous injection of 4 ml of a non-ionic contrast agent (iohexol, 300 mg/ml; Omnipaque, Amersham, Cork, Ireland), followed by 3 ml of saline solution. Resulting axial computed tomography (CT) images were transferred to an external workstation (Leonardo, Siemens Healthcare).

### Direct portography protocol

For anaesthesia, each rabbit was given an intramuscular injection of 10 mg/kg ketamine, 0.15 mg/kg of medetomidine, and 2–8 mg/kg of intravenous propofol (Propofol Lipuro®, Braun, Melsungen, Germany). The animals were placed in the supine position and a laryngeal mask size-1 was used. An 8–10 cm midline incision through the skin was made from the epigastrium for a laparotomy. Then an incision was made through the thin subcutaneous tissue to expose the linea alba. Using an inverted number-11 scalpel blade, an incision was made parallel to the linea alba. After exposure of the small bowel, a small branch of the superior mesenteric vein (SMV) was punctured with a 24-gauge needle (Abbocath, Abbott Laboratories, Chicago, IL, USA). After the stylet of the needle was removed, a 0.014-inch guidewire (Transend, Boston Scientific MediTech, Natick, MA, USA) was advanced with fluoroscopic guidance towards the liver. After removal of the cannula, a 4-F coaxial catheter introducer (Micropuncture access set, Cook Medical, USA) was advanced within the SMV. The 2-F introducer was then removed and direct portography was finally performed. Portograms were obtained with injection of 10 ml of contrast (RadialarⓇ 280 mg/ml, Juste SAQF, Madrid, Spain) into the hand. When the procedure was finished and the introducer was removed, the SMV was ligated at the level of the catheterisation. Finally the puncture site was covered with an absorbable haemostatic agent (Surgicel®, Ethicon, Somerville, NJ, USA). After confirming that there was no bleeding, the linea alba was closed in a simple continuous suture pattern with a synthetic absorbable material. The skin was closed using an interrupted pattern. Resulting images were transferred to the ImageJ programme (Rasband WS, ImageJ; National Institute of Health, Bethesda, MD, USA) where the diameter and length of the portal branches were obtained.

### Image interpretation and data collection

All CECT examinations were interpreted by two radiologists with more than 10 years of experience in hepatic CT, who selected the acquisition phase in which the original portal vein (OPV) was better demonstrated. The OPV diameter at the level immediately before the origin of the CPV was measured three times in each rabbit by each of the two radiologists. The mean of the six measurements on CECT served to calibrate and set the measurement scale for each animal. The same radiologists measured the diameter and length of the different branches of all the portograms.

**Fig. 2:**
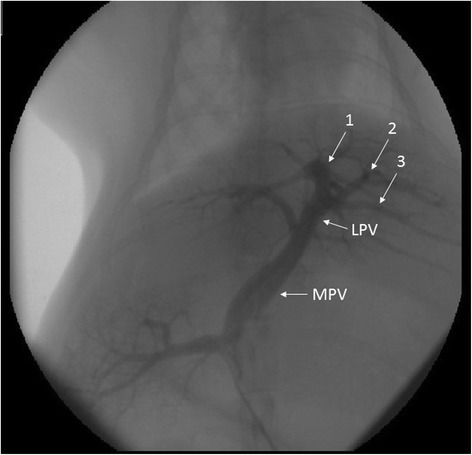
Anatomic variation type 2: trifurcation of the left portal vein (*LPV*). The LPV bifurcates into three segmental branches (*arrows 1*, *2* and *3*). *MPV* main portal vein

**Fig. 3:**
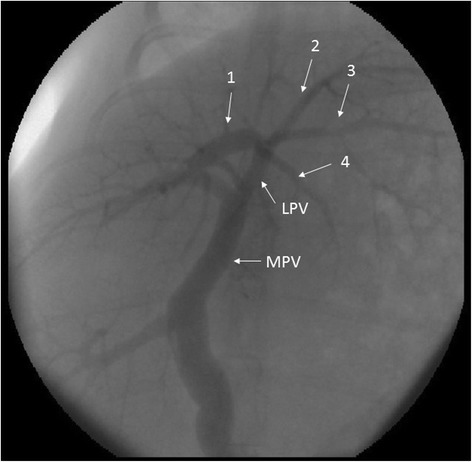
Anatomic variation type 3: quadfurcation of the left portal vein (*LPV*). The LPV bifurcates into four segmental branches (*arrows 1*, *2*, *3* and *4*). *MPV* main portal vein

**Fig. 4:**
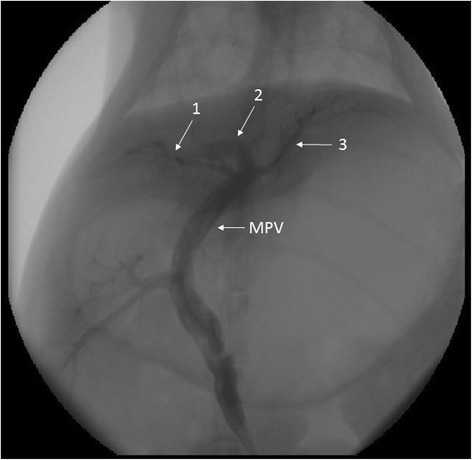
Anatomic variation type 4: trifurcation (*arrows 1*, *2* and *3*) of the main portal vein (*MPV*). In this particular case, the left portal vein and the left inferior portal vein are absent

Conventional liver rabbit portal anatomy, which was categorised as type 1, consisted of the bifurcation of the MPV into the RPV and LPV, which subsequently divided into MLPV and LLPV (Fig. 1). Any deviation from these branching variations was regarded as variant anatomy. Trifurcation of the LPV was considered type 2 anatomic variation (Fig. 2). The LPV that divides into four smaller branches was classified as type 3 (Fig. 3). Based on the origin of the LIPV, each classification type was divided in three categories: (1) if the origin was from the LPV; (2) if the origin was from the MPV; or (3) if the LIPV was absent. Other different configurations of the portal system were grouped as type 4 (Fig. 4). Because all major (lobar) portal trunks have multiple small branches (smaller than 2 mm), the length of the main trunk was obtained until there was a major bifurcation (branch bigger than 2 mm). The diameters and lengths of the following portal branches were measured: CPV, MPV, RPV, LPV, MLPV and LLPV. The angle of the OPV bifurcation (CPV and MPV) was also measured on each portogram.

**Fig. 5:**
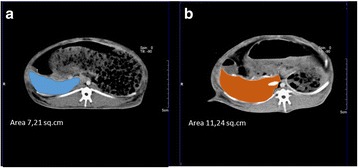
Axial views of a contrast-enhanced computed tomography scan performed in a rabbit. The volumes of the caudal lobe were calculated before (**a**) and after (**b**) radioembolisation

The caudate liver lobe and the cranial lobes were manually delineated and their respective volumes were calculated (Fig. 5).

## Results

### Contrast-enhanced CT

The measured liver volumes are shown in Table 1. The mean volume of the caudate lobe was 19.1 ml (range 8.5–33.4 ml) and of the cranial lobes it was 66.7 ml (range 26.9–88.4), and total liver volume was 85.7 ml (range 52.2–114.6) (Fig. 5).

**Table 1:** Rabbit liver volumes

	Mean volume and range (ml)	Mean volume (%)
Total liver	85.7 (52.2–114.6)	100
Cranial lobes	66.7 (26.9–88.4)	77.8
Caudate lobe	19.1 (8.5–-33.3)	22.2

### Direct portography

Table 2 summarises the frequency of the different anatomic categories. The most common portal anatomy was type 1, present in 67.4% of cases (n = 31). In this group, the MPV bifurcated to the RPV and LPV, and the latter bifurcated to the MLPV and LLPV (see Fig. 1). The origin of the LIPV in this pattern was more frequently from the LPV (Type 1a in 80.6%) than from the MPV (16.1%). The type 2 pattern, which consists of trifurcation of the LPV, was present in 15.2% of cases (n = 7) (Fig. 2). In this pattern, the LIPV originated from the LPV and MPV with the same frequency. The third most common branching pattern, LPV quadfurcation, was type 3, which appeared in 13% of cases (n = 6) (Fig. 3). The LIPV of all the rabbits of type 3 branching pattern originated from the LPV. Other anatomical variants were detected in 4.3% of cases (n = 2) and were classified as type 4. For this group, we describe two different particular cases. The first case had an MVP trifurcation directly into the RPV, the MLPV and the LLPV, without an LPV (Fig. 4). The second case consisted in an MPV bifurcation with only two lobar branches.

**Table 2:** Frequency of different portal system patterns

Patterns	Frequency
Type 1: LPV bifurcated	67.4% (*n* = 31)
Type 1a	80.6% (*n* = 25)
Type 1b	16.1% (*n* = 5)
Type 1c	3.2% (*n* = 1)
Type 2: LPV trifurcated	15.2% (*n* = 7)
Type 2a	42.9% (*n* = 3)
Type 2b	42.9% (*n* = 3)
Type 2c	14.2% (*n* = 1)
Type 3: LPV quadfurcated	13% (*n* = 6)
Type 3a	100% (*n* = 6)
Type 3b	
Type 3c	
Type 4: other	4.3% (*n* = 2)

*LPV* left portal vein

There were no variations in the portal afferents to the caudate lobe, which was always vascularised, regardless of its volume, with a unique CPV. The LIPV was present in 95.6% (44/46) of the cases and its origin was the LPV in 73.9% (36/46) and the MPV in 17.4% (8/46) of cases. The LIPV was absent in two cases.

The mean length and diameter of each main trunk are detailed in Table 3. The mean lengths before the first major bifurcation were 0.9 cm in the CPV, 1.3 cm in the RPV, 0.4 cm in the LPV, 0.6 cm in the MLPV and 0.3 cm in the LLPV. The mean diameters of each major trunk were: CPV 0.3 cm (range 0.2–0.5), RPV 0.2 cm (range 0.1–0.3), LPV 0.4 cm (range 0.3–0.7), MLPV 0.3 cm (range 0.3–0.6) and LLPV 0.2 cm 8 (range 0.1–0.4). The mean length and diameter of the MPV was 2.3 cm (range 1.1–3. 6) and 0.5 cm (range 0.4–0.9), respectively.

**Table 3:** Mean diameter and length of principal branches

Branches	Mean diameter (cm)	Mean length (cm)
MPV	0.5 (0.4–0.9)	2.3 (1.1–3.6)
CPV	0.3 (0.2–0.5)	0.9 (0.6–2.3)
RPV	0.2 (0.1–0.3)	1.3 (1.0–2.2)
LPV	0.4 (0.3–0.7)	0.4 (0.3–1.2)
MLPV	0.3 (0.3–0.7)	0.6 (0.4–1.5)
LLPV	0.2 (0.1–0.4)	0.3 (0.2–1.7)

Data in parentheses are ranges

*CPV* caudate lobe portal vein, *MPV* main portal vein, *RPV* right portal vein,

*LPV* left portal vein, *MLPV* medial left portal vein, *LLPV* lateral left portal vein

The angle between the CPV and MPV (OPV bifurcation) was 134.4° (range 97–155°).

## Discussion

Descriptive study results reveal different anatomic variations of the portal system in the rabbit liver evaluated by direct portography. We established three anatomical variants of LPV ramification which are common, and type 4 including some particular cases of MPV branching. The incidence of each type was: type 1 (67.4%), type 2 (15.2%), type 3 (13.0%) and type 4 (4.3%).

As in humans, the knowledge of the portal system anatomy and its variations is relevant when deciding technical aspects of the experimental studies. The pattern of arterial branching in the rabbit liver and its anatomical variants, and the implications on experimental designs, has been previously described by Seo and Tam [4, 5]. However, variations in the portal venous system of the liver rabbit have not been published so far. The standard description of the portal venous system only describes the conventional pattern or type 1. Some recent studies have highlighted the possibility of deploying VX2 tumoural cells within the portal vein, trying to mimic the clinical situation of liver carcinoma infiltrating the portal vein wall provoking a portal tumoural thrombus [6, 7]. In both these studies the portal vein was accessed by direct puncture and little attention is paid to the description of the portal vein anatomy. The majority of the experimental studies in rabbits use the New Zealand animal model. However, a Japanese study considered the possibility of using the Akita animal model because they are larger and thus, easier to handle [8]; the authors described the morphology of different vascular territories but did not show the portal system patterns. The need to improve knowledge on this topic is evident and is corroborated by the description of the methods used in a previous study [9] in which the classification used differs from that recommended by Seo [4].

The classification defined in the current study tries to establish the basic patterns of the portal branching of the liver rabbit. The anatomical variations were focused on the changes of the MPV. It has been obtained after a careful evaluation of a moderately large number of direct portography examinations. New studies may modify and enrich this classification as happened with Michel´s classification [10] of the hepatic arterial system in humans, which describes 10 variant subtypes with their frequency of appearance that were later modified by others such as Hiatt et al. in 1994 [11].

We also studied the volume of the whole rabbit liver and of the caudate and cranial lobes, individually. Rabbits have a relatively large caudate lobe, separated from the cranial lobes. This anatomical particularity could be useful in liver investigations, for example, into the mechanisms underlying regeneration of the normal liver.

One of the most important factors in experimental research, and specifically in liver studies, is the selection of laboratory animal species best suited for a particular purpose [12]. As an example, knowledge of details of the portal anatomy and the surrounding tissues has proven to be of utmost relevance when selecting the model for the creation of intra-hepatic or extra-hepatic portosystemic connections. The swine is an excellent model for intra-hepatic connections [13]. However, due to the presence of a liver lobe between the portal vein and the inferior vena cava, the most adequate model for extra-hepatic connections is the dog [14–16].

Our study has some limitations. First, the radiologic anatomy of the portal vein was not evaluated on CECT, because we consider direct portography more accurate to achieve this aim. Second, the portal system anatomy was evaluated only in the anteroposterior projection by two-dimensional portography.

In conclusion, we thoroughly described the conventional portal venous system anatomy of the rabbit and its variations. Because new experimental studies in rabbit liver include the portal vein access and because no homogeneous description of its morphological pattern was available, this study may offer a useful reference for experimental research planning.
